# Leapfrog/Finite Element Method for Fractional Diffusion Equation

**DOI:** 10.1155/2014/982413

**Published:** 2014-04-03

**Authors:** Zhengang Zhao, Yunying Zheng

**Affiliations:** ^1^Department of Fundamental Courses, Shanghai Customs College, Shanghai 201204, China; ^2^School of Mathematical Sciences, Huaibei Normal University, Huaibei 235000, China

## Abstract

We analyze a fully discrete leapfrog/Galerkin finite element method for the numerical solution of the space fractional order (fractional for simplicity) diffusion equation. The generalized fractional derivative spaces are defined in a bounded interval. And some related properties are further discussed for the following finite element analysis. Then the fractional diffusion equation is discretized in space by the finite element method and in time by the explicit leapfrog scheme. For the resulting fully discrete, conditionally stable scheme, we prove an *L* ^2^-error bound of finite element accuracy and of second order in time. Numerical examples are included to confirm our theoretical analysis.

## 1. Introduction

Fractional calculus and fractional partial differential equations (FPDEs) have many applications in various aspects such as in viscoelastic mechanics, power-law phenomenon in fluid and complex network, allometric scaling laws in biology and ecology, colored noise, electrode-electrolyte polarization, dielectric polarization, boundary layer effects in ducts, electromagnetic waves, quantitative finance, quantum evolution of complex systems, and fractional kinetics [1]. And a lot of attention has recently been paid to the problem of the numerical approximation of FPDEs.

Generally speaking, the finite difference method and the finite element method are the two main means to solve FPDEs. Recently, some typical fractional difference methods have been utilized to solve FPDEs numerically [2–4]. On the other hand, the finite element method has also been used to find the variational solution of FPDEs [5–14]. But there are still some interesting schemes that can be constructed to enhance the convergence order by using the finite difference/finite element mixed method.

In this paper, we use the explicit leapfrog difference/Galerkin finite element mixed method to numerically solve the space fractional diffusion equation in order to get a higher convergence order.

The fractional diffusion equation as a typical kind of fractional partial differential equation [15] is a generalization of the classical diffusion equation, which can be used to better characterize anomalous diffusion phenomena. Besides, the spatial fractional diffusion equation usually describes the Lévy flights. The operator _RL_ *D*_*a*,*x*_^2*α*^(_RL_ *D*_*x*,*b*_^2*α*^) is commonly referred to the left (right) sided Lévy stable distribution, where the underlying stochastic process is Lévy *α*-stable flights; see [16–18]. And a more general form *κ*_1_ · _RL_ *D*_*a*,*x*_^2*α*^ + *κ*_2_ · _RL_ *D*_*x*,*b*_^2*α*^ is widely used for mathematical modelling and numerical computation.

Here, we mainly focus on constructing and analyzing a kind of efficient numerical schemes for approximately solving space fractional diffusion equation. The considered problem reads as follows: for 1/2 < *α* < 1,

(1) $$\begin{array}{c} \partial_{t}u\left(x,t\right)-\Delta^{\alpha }\left(\lambda \cdot u\left(x,t\right)\right)=f\left(x,t\right),\;x\in \Omega ,\;\;t\in \left[0,T\right],\\ u\left(x,t\right)=\varphi \left(t\right),\;x\in \partial \Omega ,\;\;t\in \left[0,T\right],\\ u\left(x,0\right)=\psi \left(x\right),\;x\in \Omega , \end{array}$$

where *Ω* = [*a*, *b*], time *T* > 0. Here the spatial fractional differential operator Δ^*α*^ is denoted by *κ*_1_ · _RL_ *D*_*a*,*x*_^2*α*^ + *κ*_2_ · _RL_ *D*_*x*,*b*_^2*α*^, where 0 ≤ *κ*_1_, *κ*_2_ ≤ 1, and *κ*_1_ + *κ*_2_ = 1. When *α* = 1, the problem models a Brownian diffusion process. And *f* is a source term, *λ* is a positive constant.

The rest of this paper is constructed as follows. In Section 2, the preliminary knowledge of fractional derivative and the generalized fractional derivative spaces are defined. And some related properties are further discussed. The approximate system of the equation, existence and uniqueness of the weak solution, and the error estimates of the fully discrete scheme for (1) are studied in Section 3. In Section 4, numerical examples are presented to demonstrate the efficiency of the theoretical results derived in Section 3.

## 2. Generalized Fractional Derivative Spaces

In this section, we first give the definition of fractional derivatives. There are several definitions for the fractional derivatives, but Riemann-Liouville derivative is one of the most often used fractional derivatives, which is a reasonable generalization of the classical derivative [1, 19–22]. Then we define the generalized fractional derivative spaces by using Riemann-Liouville derivative, which is extended from the *L* ^2^ sense to the *L* ^*p*^ sense.

**Definition 1**

The *α*th order left and right Riemann-Liouville integrals of function *u*(*x*) are defined in a finite interval (*a*, *b*) as follows:

(2) DRLa,x−αu(x)=1Γ(α)∫axu(s)(x−s)1−αds,DRLx,b−αu(x)=1Γ(α)∫xbu(s)(s−x)1−αds,

where *α* > 0.

**Definition 2**

The *α*th order left and right Riemann-Liouville derivatives of function *u*(*x*) defined in a finite interval (*a*, *b*) are given as

(3) DRLa,xαu(x)=1Γ(n−α)dndxn∫ax(x−τ)n−α−1u(τ)dτ,DRLx,bαu(x)=(−1)nΓ(n−α)dndxn∫xb(τ−x)n−α−1u(τ)dτ,

in which *n* − 1 < *α* < *n* ∈ *Z* ^+^. Obviously, they are the integer derivatives of the left and right fractional integrals, respectively.

Now, we give some lemmas and corollaries which are necessary to define the generalized fractional derivative spaces.

**Lemma 3 (see [5])**

Let *Ω* = [*a*, *b*] be bounded and *α* > 0. Then *u* ∈ *L* ^*p*^(*Ω*) satisfies

(4) ||DRLa,x−αu(x)||Lp(Ω)≤(b−a)αΓ(α+1)||u||Lp(Ω),||DRLx,b−αu(x)||Lp(Ω)≤(b−a)αΓ(α+1)||u||Lp(Ω).

**Lemma 4 (fractional integration by parts, see [20])**

The relation

(5) ∫abDRLa,x−αu(x)·v(x)dx=∫abu(x)·DRLx,b−αv(x)dx

is valid under the assumption that

(6) $$\begin{array}{c} u\left(x\right)\in L^{p}\left(\Omega \right),\;\;v\left(x\right)\in L^{q}\left(\Omega \right),\\ \frac{1}{p}+\frac{1}{q}\leq 1+\alpha ,\;p\geq 1,\;\;q\geq 1, \end{array}$$

with *p* ≠ 1, *q* ≠ 1 in the case 1/*p* + 1/*q* = 1 + *α*.

**Corollary 5 (see [20])**

The formula

(7) ∫abDRLa,xαu(x)·v(x)dx=∫abu(x)·DRLx,bαv(x)dx

is valid under the assumption that *u*(*x*) ∈ _*RL*_ *D*_*a*,*x*_^−*α*^(*L* ^*p*^(*Ω*)), *v*(*x*) ∈  _*RL*_ *D*_*x*,*b*_^−*α*^(*L* ^*q*^(*Ω*)), 1/*p* + 1/*q* ≤ 1 + *α*, where the function space _*RL*_ *D*_*a*,*x*_^−*α*^(*L* ^*p*^(*Ω*)) = {*f*(*x*) | *f*(*x*) = _*RL*_ *D*_*a*,*x*_^−*α*^ *ϕ*(*x*), *ϕ*(*x*) ∈ *L* ^*p*^(*Ω*)}, _*RL*_ *D*_*x*,*b*_^−*α*^(*L* ^*q*^(*Ω*)) = {*f*(*x*) | *f*(*x*) = _*RL*_ *D*_*x*,*b*_^−*α*^ *ψ*(*x*), *ψ*(*x*) ∈ *L* ^*q*^(*Ω*)}.

**Corollary 6 (see [13])**

One can further give the following corollary:

(8) ∫abDRLa,x2αu(x)·v(x)dx=∫abDRLa,xαu(x)·DRLx,bαv(x)dx

under the assumption that *u*(*x*) ∈ _*RL*_ *D*_*a*,*x*_^−2*α*^(*L* ^*p*^(*Ω*)), *v*(*x*) ∈ _*RL*_ *D*_*x*,*b*_^−*α*^(*L* ^*q*^(*Ω*)), 1/*p* + 1/*q* ≤ 1 + *α*.

Note that the above assumption *u*(*x*) ∈ _RL_ *D*_*a*,*x*_^−2*α*^(*L* ^*p*^(*Ω*)) implies *u*(*x*) ∈ _RL_ *D*_*a*,*x*_^−*α*^(*L* ^*p*^(*Ω*)) one can prove that by using Lemma 3.

**Corollary 7 (see [13])**

Consider

(9) ∫abDRLx,b2αu(x)·v(x)dx=∫abDRLx,bαu(x)·DRLa,xαv(x)dx

under the assumption that *u*(*x*) ∈ _*RL*_ *D*_*x*,*b*_^−2*α*^(*L* ^*p*^(*Ω*)), *v*(*x*) ∈ _*RL*_ *D*_*a*,*x*_^−*α*^(*L* ^*q*^(*Ω*)), 1/*p* + 1/*q* ≤ 1 + *α*.

Note that, from the definition of the function space _RL_ *D*_*a*,*x*_^−*α*^(*L* ^*p*^(*Ω*)), we can get that if *u*(*x*) ∈ _RL_ *D*_*a*,*x*_^−*α*^(*L* ^*p*^(*Ω*)), then *u*(*x*) = _RL_ *D*_*a*,*x*_^−*α*^ *ϕ*(*x*), and _RL_ *D*_*a*,*x*_^*α*^ *u*(*x*) = *ϕ*(*x*), where *ϕ*(*x*) ∈ *L* ^*p*^(*Ω*), such that *u* ∈ *L* ^*p*^(*Ω*), which is obtained by Lemma 3. And _RL_ *D*_*a*,*x*_^*α*^ *u*(*x*) ∈ *L* ^*p*^(*Ω*) naturally holds. So, by the above idea, we define the following fractional derivative spaces from the *L* ^2^ sense to the *L* ^*p*^ sense, which will be proved to be equivalent with the fractional Sobolev spaces under some certain conditions.

**Definition 8**

Define the following norms of the left (with symbol *W*_*L*_^*α*,*p*^) fractional derivative space and the right (with symbol *W*_*R*_^*α*,*p*^) fractional derivative space in a bounded interval *Ω* = [*a*, *b*] as follows correspondingly, where 1 < *p* < +*∞*:

(10) WLα,p(Ω)≡{u∈Lp(Ω):DRLa,xαu(x)∈Lp(Ω)}

equipped with seminorm

(11) |u|WLα,p(Ω)=||Da,xαRLu(x)||Lp(Ω)

and norm

(12) $$\begin{array}{c} {||u||}_{W_{L}^{\alpha ,p}(\Omega )}={\left(\overset{\left[\alpha \right]}{\underset{k=0}{\sum }}{||D^{k}u||}_{L^{p}(\Omega )}^{p}+{\left|u\right|}_{W_{L}^{\alpha ,p}(\Omega )}^{p}\right)}^{1/p}, \end{array}$$

(13) WRα,p(Ω)≡{u∈Lp(Ω):Dx,bαRLu∈Lp(Ω)}

equipped with seminorm

(14) |u|WRα,p(Ω)=||Dx,bαRLu(x)||Lp(Ω)

and norm

(15) $$\begin{array}{c} {||u||}_{W_{R}^{\alpha ,p}(\Omega )}={\left(\overset{\left[\alpha \right]}{\underset{k=0}{\sum }}{||D^{k}u||}_{L^{p}(\Omega )}^{p}+{\left|u\right|}_{W_{R}^{\alpha ,p}(\Omega )}^{p}\right)}^{1/p}. \end{array}$$

**Definition 9**

Define the symmetric fractional derivative space (with symbol *H*_*S*_^*α*^) in a bounded interval *Ω* = [*a*, *b*] in the *L* ^2^ sense

(16) HSα(Ω)≡{u∈L2(Ω) :∫abDa,xαRLu(x)·DRLx,bαu(x)dx∈L2(Ω)}

equipped with seminorm

(17) |u|HSα(Ω)=|∫abDa,xαRLu(x)·Dx,bαRLu(x)dx|1/2

and norm

(18) $$\begin{array}{c} {||u||}_{H_{S}^{\alpha }(\Omega )}={\left(\overset{\left[\alpha \right]}{\underset{k=0}{\sum }}{||D^{k}u||}_{L^{2}(\Omega )}^{2}+{\left|u\right|}_{H_{S}^{\alpha }(\Omega )}^{2}\right)}^{1/2}. \end{array}$$

**Definition 10**

Define the spaces *W*_*L*,0_^*α*,*p*^(*Ω*), *W*_*R*,0_^*α*,*p*^(*Ω*), and *H*_*S*,0_^*α*^(*Ω*) as the closures of *C*_0_^*∞*^(*Ω*) under their respective norms.

From [6], we can get the following lemma, which is true in the *L* ^2^ sense.

**Lemma 11**

The spaces *W*_*L*,0_^*α*,2^(*Ω*), *W*_*R*,0_^*α*,2^(*Ω*), *H*_*S*,0_^*α*^(*Ω*), and *H*_0_^*α*^(*Ω*) are equal to equivalent seminorms and norms, where *H* ^*α*^(*Ω*) is the fractional Sobolev space in terms of the Fourier transform.

Therefore, in this paper we always use *H*_0_^*α*^ when *p* = 2, to denote the fractional derivative space equipped with the norm ||·||_*α*_ which can be any one of (12), (15), and (18), and *H* ^−*α*^(*Ω*) is denoted as the dual space of *H*_0_^*α*^(*Ω*), with norm ||·||_−*α*_.

Moreover, we can present some new properties about norms for the above left and right fractional derivative spaces in the *L* ^*p*^ sense.

**Lemma 12**

Let *α* > 0 and *Ω* = [*a*, *b*] ⊂ **R** be bounded. Then the following mapping properties hold:

1.   
_*RL*_ *D*_*a*,*x*_^−*α*^ *u*(*x*) : *L* ^*p*^(*Ω*) → *L* ^*p*^(*Ω*) is a bounded linear operator;
2.   
_*RL*_ *D*_*x*,*b*_^−*α*^ *u*(*x*) : *L* ^*p*^(*Ω*) → *L* ^*p*^(*Ω*) is a bounded linear operator;
3.   
_*RL*_ *D*_*a*,*x*_^*α*^ *u*(*x*) : *W*_*L*_^*α*,*p*^(*Ω*) → *L* ^*p*^(*Ω*) is a bounded linear operator;
4.   
_*RL*_ *D*_*x*,*b*_^*α*^ *u*(*x*) : *W*_*R*_^*α*,*p*^(*Ω*) → *L* ^*p*^(*Ω*) is a bounded linear operator;
5.   
_*RL*_ *D*_*a*,*x*_^−*α*^ *u*(*x*) : *L* ^*p*^(*Ω*) → *W*_*L*_^*α*,*p*^(*Ω*) is a bounded linear operator;
6.   
_*RL*_ *D*_*x*,*b*_^−*α*^ *u*(*x*) : *L* ^*p*^(*Ω*) → *W*_*R*_^*α*,*p*^(*Ω*) is a bounded linear operator.

**Proof**

Properties (1) and (2) follow directly from Lemma 3.

Property (3) follows directly from the definition of *W*_*L*_^*α*,*p*^(*Ω*) and *W*_*R*_^*α*,*p*^(*Ω*) as

(19) ||Da,xαRLu(x)||Lp(Ω)  ≤(∑k=0[α]||Dku||Lp(Ω)p+||DRLa,xαu(x)||Lp(Ω)p)1/p.

Property (4) follows similarly.

Property (5) follows from the definition of *W*_*L*_^*α*,*p*^(*Ω*) and the semigroup property of fractional operator,

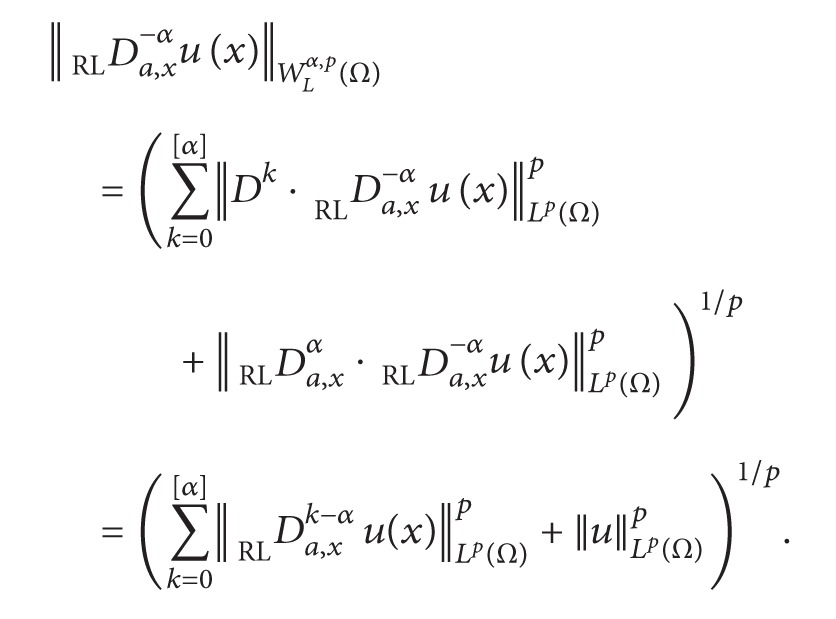
(20)
Using Lemma 3, there exist constants *c*_*k*_, *k* = 0,…, [*α*] such that

(21) ||Da,xk−αRLu(x)||Lp(Ω)p≤Ck||u||Lp(Ω)p.

Therefore, we obtain the bound

(22) ||Da,x−αRLu(x)||WLα,p(Ω)≤(1+∑k=0[α]ck)1/p||u||Lp(Ω).

Property (6) follows similarly.

**Corollary 13**

Consider

(23) $$\begin{array}{c} W_{L,0}^{\alpha ,p}\left(\Omega \right)⟶W_{L,0}^{\alpha -[\alpha ],p}\left(\Omega \right),\\ W_{R,0}^{\alpha ,p}\left(\Omega \right)⟶W_{R,0}^{\alpha -[\alpha ],p}\left(\Omega \right) \end{array}$$

for 1 ≤ *p* < *∞*. And if 1 ≤ *p* ≤ *q* < *∞*, one has

(24) $$\begin{array}{c} W_{L,0}^{\alpha ,p}\left(\Omega \right)⟶W_{L,0}^{\alpha ,q}\left(\Omega \right),\\ W_{R,0}^{\alpha ,p}\left(\Omega \right)⟶L^{q}\left(\Omega \right). \end{array}$$

It is obviously true by using the norms of fractional derivative spaces and imbedding theorems for *L* ^*p*^(*Ω*).

**Lemma 14**

Let *Ω* = [*a*, *b*] ⊂ **R** be bounded. Then for *u* ∈ *W*_*L*,0_^*α*,*p*^(*Ω*), one has

(25) $$\begin{array}{c} \begin{array}{c} {||u||}_{L^{p}(\Omega )}\leq C{||u||}_{W_{L,0}^{\alpha ,p}(\Omega )}, \end{array} \end{array}$$

and for 0 < *s* < *α*, one has

(26) $$\begin{array}{c} {||u||}_{W_{L,0}^{s,p}(\Omega )}\leq C{||u||}_{W_{L,0}^{\alpha ,p}(\Omega )}. \end{array}$$

**Proof**

If *u* ∈ *W*_*L*,0_^*α*,*p*^(*Ω*) by using Lemmas 3 and 12, we have that

(27) ||u||Lp(Ω)=||Da,x−αRL DRLa,xαu(x)||Lp(Ω)≤(b−a)αΓ(α+1)||DRLa,xαu(x)||Lp(Ω).

Therefore, (25) is true. And the following inequality certainly holds:

(28) ||u||Lp(Ω)≤C||DRLa,xα−su(x)||Lp(Ω).

So, we get that

(29) ||DRLa,xsu(x)||Lp(Ω)≤||DRLa,xs DRLa,xα−su(x)||Lp(Ω)=||DRLa,xαu(x)||Lp(Ω).

Therefore, (26) holds.

## 3. Error Estimates of the Leapfrog/Finite Element Scheme

In this section, we firstly give a fully discrete scheme, where we use the leapfrog difference method in the temporal direction and the finite element method in the spatial direction and then analyze the error estimate. Let *S*_*h*_ denote a uniform partition on *Ω*, with grid parameter *h*. For *k* ∈ *N*, let *P*_*k*_(*Ω*) denote the space of polynomials on *Ω* with degree not greater than *k*. Then we define *X*_*h*_ as the finite element space on *S*_*h*_ with the basis of the piecewise polynomials of order *k* ∈ *Z* ^+^; that is,

(30) $$\begin{array}{c} X_{h}=\left\{v\in X\cap C\left(\bar{\Omega }\right):v{\;|\;}_{D}\in P_{k}\left(D\right),\forall D\in S_{h}\right\}, \end{array}$$

in which *D* is the unit of *S*_*h*_.

The following property of finite element spaces is necessary for our subsequent analysis [23]: for *u* ∈ *H* ^*k*+1^(*Ω*), 0 ≤ *μ* ≤ *k* + 1, there exists *v* ∈ *X*_*h*_ such that

(31) $$\begin{array}{c} {||u-v||}_{\mu }\leq Ch^{k+1-\mu }{||u||}_{k+1}. \end{array}$$

The Gronwall's lemma is also needed for the error analysis.

**Lemma 15 (discrete Gronwall's lemma, see [24])**

Let Δ*t*, *H* and *a*_*n*_, *b*_*n*_, *c*_*n*_, *γ*_*n*_ (for integer *n* ≥ 0) be nonnegative numbers such that

(32) $$\begin{array}{c} a_{N}+\Delta t\overset{N}{\underset{n=0}{\sum }}b_{n}\leq \Delta t\overset{N}{\underset{n=0}{\sum }}\gamma_{n}a_{n}+\Delta t\overset{N}{\underset{n=0}{\sum }}c_{n}+H, \end{array}$$

for *N* ≥ 0. Suppose that Δ*tγ*_*n*_ < 1 for all *n*, and set *σ*_*n*_ = (1 − Δ*tγ*_*n*_)^−1^; then

(33) $$\begin{array}{c} a_{N}+\Delta t\overset{N}{\underset{n=0}{\sum }}b_{n}\leq \exp\left(\Delta t\overset{N}{\underset{n=0}{\sum }}\sigma_{n}\gamma_{n}\right)\left\{\Delta t\overset{N}{\underset{n=0}{\sum }}c_{n}+H\right\} \end{array}$$

for *N* ≥ 0.

In the following, we give the fully discrete scheme of (1). Let Δ*t* denote the step size for *t* so that *t*_*n*_ = *n*Δ*t*, *n* = 1,2,…, *N* − 1. For notational convenience, we denote *u* ^*n*^ : = *u*(·, *t*_*n*_) and

(34) $$\begin{array}{c} d_{t}u^{n}:=\frac{u^{n+1}-u^{n-1}}{2\Delta t}. \end{array}$$

Let *u*_*h*_^*n*^ of (1) be the finite element solution at time *t* = *t*_*n*_ of the following fully discrete scheme:

(35) $$\begin{array}{c} \begin{array}{ccc} \left(d_{t}u_{h}^{n},v\right)-\left(\Delta^{\alpha }\left(\lambda \cdot u_{h}^{n}\right),v\right)=\langle f^{n},v\rangle ,\;\forall v\in X_{h}; & & \end{array} \end{array}$$

that is,

(36) (uhn+1−uhn−1,v)−2Δt(Δα(λ·uhn),v)  =2Δt〈fn,v〉, ∀v∈Xh,

where (·, ·) is denoted by an *L* ^2^ inner product and (Δ^*α*^(*λ* · *u*_*h*_^*n*^), *v*) = *κ*_1_ · (_RL_ *D*_*a*,*x*_^*α*^(*λ* · *u*_*h*_^*n*^), _RL_ *D*_*x*,*b*_^*α*^ *v*) + *κ*_2_ · (_RL_ *D*_*x*,*b*_^*α*^(*λ* · *u*_*h*_^*n*^), _RL_ *D*_*a*,*x*_^*α*^ *v*). For brevity, we always use (Δ^*α*^(*λ* · *u*_*h*_^*n*^), *v*) instead of the right hand side of this equation.

**Lemma 16**

For a sufficient small step size Δ*t* > 0, there exists a unique solution *u*_*h*_^*n*^ ∈ *X*_*h*_ satisfying (36).

**Proof**

Firstly, we prove that (*u*_*h*_^*n*^, *u*_*h*_^*n*^)/2Δ*t* − (Δ^*α*^(*λ* · *u*_*h*_^*n*^), *u*_*h*_^*n*^) is positive, which is one of the sufficient conditions for the existence and uniqueness of *u*_*h*_^*n*^.

For Δ*t* > 0 chosen sufficiently small, we have that

(37) $$\begin{array}{c} \frac{\left(u_{h}^{n},u_{h}^{n}\right)}{2\Delta t}-\left(\Delta^{\alpha }\left(\lambda \cdot u_{h}^{n}\right),u_{h}^{n}\right)\geq C{||u_{h}^{n}||}_{\alpha }^{2}. \end{array}$$

Besides, by using the fractional Poincare-Friedrichs formula, we can easily get the continuity of (*u*_*h*_^*n*^, *u*_*h*_^*n*^)/2Δ*t* − (Δ^*α*^(*λ* · *u*_*h*_^*n*^), *u*_*h*_^*n*^). Hence, by using the Lax-Milgram theorem, we have that (36) is uniquely solvable for *u*_*h*_^*n*^.

Now, we carry out the error analysis for the fully discrete problem. The following norms are also used in the analysis:

(38) $$\begin{array}{c} {||u||}_{\infty ,k}=\underset{1\leq n\leq N}{\max}{||u^{n}||}_{k},\\ {||u||}_{0,\alpha }={\left(\overset{N}{\underset{n=1}{\sum }}{||u^{n}||}_{\alpha }^{2}dt\right)}^{1/2}. \end{array}$$

**Theorem 17**

Assume that (1) has a solution *u* satisfying *u* ∈ *L* ^2^(0, *T*; *H* ^*α*^∩*H* ^*k*+1^(*Ω*)), *u*_*t*_ ∈ *L* ^2^(0, *T*; *H* ^*k*+1^(*Ω*)), and *u*_*ttt*_ ∈ *L* ^2^(0, *T*; *L* ^2^(*Ω*)), with *u* ^0^ ∈ *H* ^*k*+1^(*Ω*). *u*_*h*_^*n*^ is the solution of (36), and *u*_*h*_^1^ is computed in such a way that

(39) $$\begin{array}{c} ||u^{1}\left(x\right)-u\left(x,\Delta t\right)||\leq C{\left(\Delta t\right)}^{2}. \end{array}$$

Then, there exists a constant *C*_0_ independent of *h* and Δ*t*, such that if

(40) $$\begin{array}{c} \Delta t\cdot h^{-2\alpha }\leq C_{0}, \end{array}$$

then the finite element approximation (36) is convergent to the solution of (1) on the interval (0, *T*) as Δ*t*, *h* → 0. And the approximation solution *u*_*h*_ satisfies the following error estimates:

(41) ||u−uh||0,α≤C(hk+1||ut||0,k+1+(Δt)2||uttt||0,0   +hk+1−α||u||0,k+1);

(42) ||u−uh||∞,0≤C(hk+1||ut||0,k+1+(Δt)2||uttt||0,0   +hk+1−α||u||0,k+1+hk+1||u||∞,k+1).

**Proof**

In order to estimate (41) and (42), we first discuss the error at *t* = *t*_*n*_,  *n* = 1,2,…, *N* − 1. Let *u* ^*n*^ = *u*(·, *t*_*n*_) represent the solution of (1), define *ɛ* ^*n*^ = *u* ^*n*^ − *u*_*h*_^*n*^, and for *U* ^*n*^ ∈ *X*_*h*_, define Λ^*n*^ and *E* ^*n*^ as Λ^*n*^ = *u* ^*n*^ − *U* ^*n*^, *E* ^*n*^ = *U* ^*n*^ − *u*_*h*_^*n*^. So, we have *ɛ* ^*n*^ = Λ^*n*^ + *E* ^*n*^.

Obviously the true solution of this problem (1) *u* ^*n*^ also satisfies

(43) (dtun,v)−(Δα(λ·un),v) =〈fn,v〉−(utn−dtun,v), ∀v∈Xh.

Therefore, subtracting (36) from (43) gives

(44) $$\begin{array}{c} \left(d_{t}\epsilon^{n},v\right)-\left(\Delta^{\alpha }\left(\lambda \cdot \epsilon^{n}\right),v\right)=\left(d_{t}u^{n}-u_{t}^{n},v\right),\;\forall v\in X_{h}; \end{array}$$

that is,

(45) (ϵn+1−ϵn−1,v)−2Δt(Δα(λ·ϵn),v) =2Δt(dtun−utn,v), ∀v∈Xh.

Substituting *ϵ* ^*n*+1^ = Λ^*n*+1^ + *E* ^*n*+1^, *v* = *E* ^*n*+1^ + *E* ^*n*−1^ into (45) leads to

(46) (En+1−En−1,En+1+En−1)  −2Δt(Δα(λ·En),En+1+En−1) =−(Λn+1−Λn−1,En+1+En−1)  +2Δt(Δα(λ·Λn),En+1+En−1)  +2Δt(dtun−utn,En+1+En−1).

After adding ||*E* ^*n*^||^2^ to both sides of (46), we obtain the identity

(47) ||En+1||2+||En||2−2Δt(Δα(λ·En),En+1) =||En||2+||En−1||2+2Δt(Δα(λ·En),En−1)  −(Λn+1−Λn−1,En+1+En−1)  +2Δt(Δα(λ·Λn),En+1+En−1)  +2Δt(dtun−utn,En+1+En−1).

Define now the quantity *A* ^*n*+1^, for 1 ≤ *n* ≤ *N* − 1, by

(48) $$\begin{array}{c} A^{n+1}={||E^{n+1}||}^{2}+{||E^{n}||}^{2}-2\Delta t\left(\Delta^{\alpha }\left(\lambda \cdot E^{n}\right),E^{n+1}\right). \end{array}$$

We can rewrite (47) as

(49) An+1=An−(Λn+1−Λn−1,En+1+En−1) +2Δt(Δα(λ·En),En−1) +2Δt(Δα(λ·Λn),En+1+En−1) +2Δt(Δα(λ·En−1),En) +2Δt(dtun−utn,En+1+En−1).

Denoting

(50) F(En−1,En,En+1)=−(Λn+1−Λn−1,En+1+En−1)+2Δt(Δα(λ·En),En−1)+2Δt(Δα(λ·Λn),En+1+En−1)+2Δt(Δα(λ·En−1),En)+2Δt(dtun−utn,En+1+En−1),

then (49) can be abbreviated as

(51) $$\begin{array}{c} A^{n+1}=A^{n}+F\left(E^{n-1},E^{n},E^{n+1}\right). \end{array}$$

We now estimate each term in *F*(*E* ^*n*−1^, *E* ^*n*^, *E* ^*n*+1^). For the second term of the right hand side, one has

(52) 2Δt(Δα(λ·En),En−1) =2Δt(κ1(DRLa,xα(λ·En),DRLx,bαEn−1)+κ2(DRLx,bα(λ·En),DRLa,xαEn−1)) ≤C1Δt||En||α2+C2Δt||En−1||α2,(Λn+1−Λn−1,En+1+En−1) ≤||Λn+1−Λn−1||·||En+1+En−1|| =2Δt||dtΛn||·||En+1+En−1|| ≤Δt||dtΛn||2+Δt(||En+1||2+||En−1||2) ≤C3Δt·h2k+2||utn||k+12+Δt(||En+1||2+||En−1||2),

where

(53) ∑n=0NΔt||dtΛn||2 =∑n=1NΔt||1Δt∫tn−1tn1∂Λ∂tdt||2 ≤∑n=1NΔt(1Δt)2∫Ω(∫tn−1tn1dt)(∫tn−1tn∂Λ∂tdt)dx ≤C3h2k+2||ut||0,k+12.

For the third term of the right hand side, one has

(54) 2Δt(Δα(λ·Λn),En+1+En−1) =2Δt·κ1(DRLa,xα(λ·Λn),DRLx,bα(En+1+En−1))  +2Δt·κ2(DRLx,bα(λ·Λn),DRLa,xα(En+1+En−1)) ≤C4Δt||Λn||α·||En+1+En−1||α ≤C5Δt·h2(k+1−α)||un||k+12  +C6Δt(||En+1||α2+||En−1||α2),

in which

(55) $$\begin{array}{c} {||\lambda \cdot \Lambda^{n}||}_{\alpha }\leq {||\lambda ||}_{\infty }\cdot {||\Lambda^{n}||}_{\alpha }\leq C_{5}h^{k+1-\alpha }{||u^{n}||}_{k+1}. \end{array}$$

For the fourth term of the right hand side, one has

(56) 2Δt(Δα(λ·En−1),En) =2Δt·κ1(DRLa,xαλ·En−1,DRLx,bαEn)  +2Δt·κ2(DRLx,bαλ·En−1,DRLa,xαEn) ≤C7Δt·||En−1||α2+C8Δt·||En||α2.

And for the term 2Δ*t*(*d*_*t*_ *u* ^*n*^ − *u*_*t*_^*n*^, *E* ^*n*+1^ + *E* ^*n*−1^), by using the Cauchy-Schwarz inequality, we obtain

(57) 2Δt(dtun−utn,En+1+En−1) =(un+1−un−1−2Δt·utn,En+1+En−1) ≤C9(Δt)5||utttn||2+Δt(||En+1||2+||En−1||2),

where by Taylor's theorem

(58) $$\begin{array}{c} ||u^{n+1}-u^{n-1}-2\Delta t\cdot u_{t}^{n}||\leq C_{9}{\left(\Delta t\right)}^{3}||u_{ttt}^{n}||. \end{array}$$

Hence, summing from *n* = 1 to *N* − 1, one has

(59) $$\begin{array}{c} A^{N}-A^{N-1}\leq \overset{N-1}{\underset{n=1}{\sum }}F\left(E^{n-1},E^{n},E^{n+1}\right); \end{array}$$

that is,

(60) AN≤AN−1 +C10∑n=1N−1Δt(h2k+2||utn||k+12+h2k+2−2α||un||k+12+(Δt)4||utttn||2) +C11∑n=1N−1Δt(||En+1||2+||En−1||2+||En||α2+ ||En−1||α2+||En+1||α2).

We now show that, under our stability assumption (40), *A* ^*n*+1^ is positive and comparable to ||*E* ^*n*^||^2^ + ||*E* ^*n*+1^||^2^. To this end, we use the inverse inequality ||*v*||_*α*_ ≤ *C*_12_ *h* ^−*α*^||*v*||, *v* ∈ *X*_*h*_, and this yields

(61) 2Δt|−(Δα(λ1En,En+1))| ≤Δt(||En||α2+||En+1||α2) ≤C12Δt·h−2α(||En||2+||En+1||2).

Hence, if Δ*t* · *h* ^−2*α*^ is sufficiently small such that *C*_12_Δ*t* · *h* ^−2*α*^ ≤ *C*_13_ ≤ 1, we get

(62) (1−C13)(||EN−1||2+||EN||2) ≤AN≤(1+C13)(||EN−1||2+||EN||2).

So we have that

(63) ||EN−1||2+||EN||2+C14∑n=1NΔt||En||α2 ≤||E1||2+||E0||2  +C15∑n=1N−1Δt(h2k+2||utn||k+12+h2k+2−2α||un||k+12+(Δt)4||utttn||2)  +C16∑n=1N−1Δt(||En+1||2+||En−1||2).

Therefore, we obtain

(64) ||EN−1||2+||EN||2+C14∑n=1NΔt||En||α2 ≤||E1||2+||E0||2  +C15(h2k+2||ut||0,k+12+h2k+2−2α||un||k+12+(Δt)4||uttt||0,02)  +C16∑n=1N−1Δt(||En+1||2+||En−1||2).

Hence,

(65) ||EN||2+C14∑n=1NΔt||En||α2 ≤||E1||2+||E0||2  +C15(h2k+2||ut||0,k+12+h2k+2−2α||un||k+12+(Δt)4||uttt||0,02)+C16∑n=1N−1Δt||En+1||2.

By using the discrete Gronwall's Lemma 15, we have

(66) ||EN||2+C14∑n=1NΔt||En||α2 ≤C17||E1||2  +C15(h2k+2||ut||0,k+12+h2k+2−2α||u||0,k+12+(Δt)4||uttt||0,02),

where denoting *a*_*n*_ = ||*E* ^*N*^||^2^, *b*_*n*_ = ||*E* ^*n*^||_*α*_^2^, *H* = *C*_15_(*h* ^2*k*+2^||*u*_*t*_||_0,*k*+1_^2^ + *h* ^2*k*+2−2*α*^||*u* ^*n*^||_*k*+1_^2^ + (Δ*t*)^4^||*u*_*ttt*_||_0,0_^2^).

Now denoting *G*(Δ*t*, *h*) = *h* ^2*k*+2^||*u*_*t*_||_0,*k*+1_^2^ + *h* ^2*k*+2−2*α*^||*u*||_0,*k*+1_^2^ + (Δ*t*)^4^||*u*_*ttt*_||_0,0_^2^ and using the condition (39), we get that

(67) $$\begin{array}{c} {||E||}_{0,\alpha }^{2}=\overset{N}{\underset{n=1}{\sum }}\Delta t{||E^{n}||}_{\alpha }^{2}\leq C_{18}\left(T+1\right)G\left(\Delta t,h\right). \end{array}$$

By using the interpolation property and the following result

(68) $$\begin{array}{c} {||u-u_{h}||}_{0,\alpha }\leq {||E||}_{0,\alpha }+{||\Lambda ||}_{0,\alpha }, \end{array}$$

estimate (41) follows.

Using estimate (66) and approximation properties, we have

(69) ||u−uh||∞,02≤||E||∞,02+||Λ||∞,02≤G(Δt,h)+h2k+2||u||∞,k+12,

which yields estimate (42).

## 4. Numerical Examples for Piecewise Linear Polynomials

Let *S*_*h*_ denote a uniform partition on *Ω* = [*a*, *b*] and *X*_*h*_ the space of continuous piecewise linear functions on *S*_*h*_; that is, *k* = 1. Then we use the Galerkin finite element method for the spatial variables. After the spatial discretization, we get classical ODEs systems with variables *u*_*h*_^*i*^, *i* = 1,2,…, *T*/Δ*t*. In order to satisfy the condition (39) in Theorem 17, we use the two-order Runge-Kutta method to compute the variable *u*_*h*_^1^.

In this section, we present numerical calculations which support the error estimates in Theorem 17. If we suppose Δ*t* = *Ch* ^2*α*^, then we have the convergence rate

(70) $$\begin{array}{c} {||u(t_{n+1})-u_{h}^{n+1}||}_{0,\alpha }\sim 𝒪\left(h^{2-\alpha }\right),\\ {||u(t_{n+1})-u_{h}^{n+1}||}_{\infty ,0}\sim 𝒪\left(h^{2-\alpha }\right). \end{array}$$

**Example 1**

(i) Let

(71) $$\begin{array}{c} u\left(x,t\right)=t^{2}x\left(1-x\right) \end{array}$$

then *u* is the exact solution to the problem

(72) $$\begin{array}{c} \partial_{t}u\left(x,t\right)-\Delta^{\alpha }\left(\lambda \cdot u\left(x,t\right)\right)=f\left(x,t\right),\;x\in \Omega ,\;\;t\in \left[0,T\right],\\ u\left(0,t\right)=u\left(1,t\right)=0,\;t\in \left[0,T\right],\\ u\left(x,0\right)=0,\;x\in \Omega , \end{array}$$

where *α* = 0.8, *κ*_1_ = *κ*_2_ = 1/2, *λ* = 1, *Ω* = [0,1], *T* = 1, and

(73) f(x,t)=2tx(1−x) −t2(x1−2α+(1−x)1−2α2Γ(2−2α)+x2−2α+(1−x)2−2α2Γ(3−2α)).

The experiential error results and convergence rates are presented in Table 1.

(ii) Let

(74) $$\begin{array}{c} u\left(x,t\right)=e^{-t}x\left(x+1\right) \end{array}$$

be the exact solution to the problem

(75) $$\begin{array}{c} \partial_{t}u\left(x,t\right)-\Delta^{\alpha }\left(\lambda \cdot u\left(x,t\right)\right)=f\left(x,t\right),\;x\in \Omega ,\;\;t\in \left[0,T\right],\\ u\left(0,t\right)=0,u\left(1,t\right)=2e^{-t}\;t\in \left[0,T\right],\\ u\left(x,0\right)=x\left(x+1\right),\;x\in \Omega , \end{array}$$

where *α* = 0.6, *κ*_1_ = *κ*_2_ = 1/2, *λ* = 1, *Ω* = [0,1], *T* = 1, and  *f*(*x*, *t*) is numerically obtained.

The experiential error results and convergence rates are displayed in Table 2.

## 5. Conclusion

In this paper, we study the finite element method for fractional diffusion equation. We use the simple, second order accurate explicit scheme, leapfrog difference method in time, and the finite element method in space. Under the suitably accurate initial conditions and the stability requirement that Δ*t* · *h* ^−2*α*^ be sufficiently small, the error analysis for the fully discrete scheme is discussed, which is an *L* ^2^-error bound of finite element accuracy and of second order in time. Numerical examples are given to demonstrate the efficiency of the theoretical results.

## Figures and Tables

**Table 1:** The experiential error results and convergence rates of Example 1 (i).

*h*	||*u*−*u*_*h*_||_0,0_	cv. rate	||*u*−*u*_*h*_||_∞,0_	cv. rate
1/4	1.0569 · 10^−2^	—	3.1077 · 10^−2^	—

1/8	4.0416 · 10^−3^	1.3869	6.5469 · 10^−3^	2.2470

1/16	3.8027 · 10^−4^	3.4098	4.4162 · 10^−4^	3.8899

1/32	1.0910 · 10^−4^	1.8014	1.4097 · 10^−4^	1.6474

1/64	4.8599 · 10^−5^	1.1551	6.4241 · 10^−5^	1.1338

**Table 2:** The experiential error results and convergence rates of Example 1 (ii).

*h*	||*u*−*u*_*h*_||_0,0_	cv. rate	||*u*−*u*_*h*_||_∞,0_	cv. rate
1/4	5.6283 · 10^−3^	—	9.3921 · 10^−3^	—

1/8	1.9379 · 10^−3^	1.5382	3.2521 · 10^−3^	1.5301

1/16	7.1701 · 10^−4^	1.4344	1.2910 · 10^−3^	1.4274

1/32	2.6932 · 10^−4^	1.4128	4.7917 · 10^−4^	1.3353

1/64	1.0362 · 10^−4^	1.3780	1.9584 · 10^−4^	1.2909
